# Assessment and predictors of knowledge and attitudes of women toward breastfeeding in the Kurdistan Region, Iraq: a cross-sectional study

**DOI:** 10.1186/s12889-026-28031-7

**Published:** 2026-06-02

**Authors:** Sharmin Abdalrahman Karim, Sumaya Ali Hamad, Awin Mahmud Abdulrahman

**Affiliations:** College of Nursing, University of Raparin, Rania, Sulaymaniyah, Kurdistan Region Iraq

**Keywords:** Age, Attitude, Breastfeeding, Infant nutrition, Knowledge, Positive predictors

## Abstract

**Background:**

Breastfeeding practices in Iraq, especially in the Kurdistan Region, remain below global recommendations that might lead to negative consequences. So, the aim of this study was to assess breastfeeding knowledge and attitudes among Kurdish mothers and identify main demographic predictors.

**Methods:**

A descriptive cross-sectional study was conducted on 412 Kurdish mothers who had breastfed at least one child, recruited from public and private maternal healthcare facilities across the Kurdistan Region, Iraq, from December 2024 to February 2025. Following ethical approval from the University Ethical Committee, data were collected via a structured and validated questionnaire to assess breastfeeding knowledge (11 dichotomous items) and attitudes (8 Likert-scale items). Then, ordinal logistic regression was used to identify significant predictors.

**Results:**

The mean age of the participants was 30.4 ± 7.1 years. Only 20.6% and 18.7% of mothers demonstrated high breastfeeding knowledge and positive attitude, respectively. Younger mothers aged ≤ 24 (Aor = 0.430; 95% CI: 0.187–0.985; *p* = 0.046) and 25–32 years (Aor = 0.462; 95% CI: 0.234–0.910; *p* = 0.025) were less likely to have high knowledge than those ≥ 41 years. Illiterate women had markedly lower odds of high knowledge (Aor = 0.280; *p* = 0.016) and more likely positive attitude (Aor = 3.567; *p* = 0.041) than those hold postgraduate certificates. Receiving breastfeeding education from relatives was significantly associated with lower odds of a positive attitude (Aor = 0.345; 95% CI: 0.167–0.715; *p* = 0.004), whereas a monthly income of 400,000–600,000 IQD had a positive association (COR = 1.972; 95% CI: 1.049 − 3.707; *p* = 0.035).

**Conclusion:**

Younger and less-educated mothers exhibit poorer breastfeeding knowledge and attitudes. Thus targeted health education is essential to improve breastfeeding outcomes in the region.

## Introduction

Breastfeeding is recognized as the optimal source of infant nutrition and a cornerstone of child survival. Breast milk contains antibodies, hormones and immune factors to protect the baby against common infections [1, 2]. World Health Organization (WHO) and UNICEF emphasize its benefits for both child and mother, as it reduces risks of infant mortality and later obesity or diabetes in breastfed children, and lower breast and ovarian cancer risks in mothers [3, 4]. However, globally, fewer than half of infants aged < 6 months are exclusively breastfed [5, 6] and about 46% begin breastfeeding within an hour of birth.

The WHO recommend that newborns be placed skin-to-skin and put to the breast within the first hour of life, be fed only breast milk for 6 months, and continue breastfeeding up to ≥ 2 years with complementary foods introduced after 6 months [7]. Evidence-based campaigns and policies have been adopted in many countries to support this; however, international surveys show that uptake of these recommendations remains suboptimal, as only about half of women worldwide adhere to exclusive breastfeeding for the full 6 months. While exclusive breastfeeding rates have risen by about 10% over the past decade, reaching ~ 48% globally [8]. In 2023, UNICEF and WHO reported that exclusive breastfeeding rates have only reached ~ 48% worldwide, still just shy of the WHO’s 2025 target of 50% [8]. Many regions, including the Middle East still report exclusive breastfeeding well below targets. In Iraq, infant nutrition indicators are concerning, as ~ 20% of children aged < 5 years are stunted, reflecting chronic malnutrition, and UNICEF reports that only 19.6% of infants are exclusively breastfed immediately after birth. Continuation of breastfeeding is also poor in Iraq, as 33% of newborns were breastfed within the first hour of life, 25% were exclusively breastfed for 6 months, and 22.7% breastfed for one year. These low rates occur in a context of ongoing conflict and displacement, which UNICEF notes further jeopardize child feeding and nutrition [9]. The Kurdistan Region of Iraq shares many of these challenges with low adherence of mothers to optimal breastfeeding in the first 6 months, as reported in Sulaimaniyah (46.5%) [10] and Duhok (8%) [11]. These figures are below WHO targets and the modest national average. Kurdish mothers often receive breastfeeding-related information from elder female relatives, particularly grandmothers and mothers-in-law, whose traditional beliefs may influence maternal feeding decisions. Informal advice from relatives may sometimes override professional healthcare recommendations. The Kurdish sociocultural environment differs from many neighboring regions because maternal and infant care practices are frequently shaped by extended family systems, cultural expectations, and traditional postpartum practices. These contextual factors may influence breastfeeding knowledge, attitudes, and continuation. Significant gaps and barriers persist, especially in low and middle-income regions. Thus, despite strong guidelines, compliance with breastfeeding recommendations in developing countries is low [12, 13]. Although breastfeeding practices have been investigated in some Iraqi settings, comprehensive evidence regarding breastfeeding knowledge and attitudes among Kurdish mothers remains limited. Understanding these factors within the unique sociocultural and post-conflict healthcare context of the Kurdistan Region is important for developing culturally appropriate maternal and child health interventions. Consequently, in this locality, no comprehensive cross-sectional data exist on this aspect. To address this literature gap, this study addresses the following fundamental research questions: What is the current level of breastfeeding knowledge and attitudes among Kurdish mothers in Iraq? and what are the primary sociodemographic and cultural factors that predict these outcomes? By answering this question, this study aimed to assess Kurdish women’s knowledge and attitudes toward breastfeeding and their predictors.

##  Methods

### Study design and setting

This quantitative, descriptive, cross-sectional study was conducted on 412 mothers from December 2024 to February 2025, in public and private maternity hospitals and primary healthcare centers in different areas of Iraqi Kurdistan Region, including Erbil, Sulaymaniyah, Halabja, Koya, Rania and Qaladze to ensure comprehensive representation of urban, semi-urban, and peripheral populations.

### Inclusion criteria

Kurdish mothers who aged ≥ 18 years and had breastfed at least one child.

### Exclusion criteria

Mothers with significant communication barriers or cognitive impairments that could interfere with accurate completion of the questionnaire.

### Sampling method and sample size

A non-probability purposive sampling strategy was employed to recruit eligible participants from public and private maternal healthcare facilities. This approach was scientifically justified because the study required an information-rich sample meeting strict clinical and experiential criteria (i.e., Kurdish mothers who had actively breastfed at least one child). Given the specific target population and the facility-based recruitment framework, purposive sampling ensured optimal feasibility, efficient resource allocation, and direct access to eligible participants who could accurately provide data on localized breastfeeding experiences and sociocultural influences. While we acknowledge that non-probability sampling limits broader statistical generalizability across the entire national population, it was the most robust and practical mechanism to satisfy our specific inclusion criteria and capture deep insights into local maternal attitudes. Thus, the required sample size was determined using Cochran’s formula for cross-sectional studies, assuming 95% confidence level, 5% margin of error, and an estimated population proportion of 50% to ensure maximum variability [14]. This calculation yielded a minimum sample size of 384 participants. To enhance statistical power and compensate for potential non-response or data inconsistencies, a total of 412 mothers were ultimately enrolled in the study.

### Study tools and measures

Data were gathered using a structured, validated questionnaire adapted from previous research [15]. The instrument was reviewed by a multidisciplinary panel of experts in maternal and child health, nursing, paediatrics, public health, and biostatistics to ensure content relevance, clarity, and contextual appropriateness. The questionnaire comprised three primary sections. The first section captured sociodemographic and clinical information, including maternal age, level of education, place of residence, employment status, family income, parity, current pregnancy status, and history of antenatal care. The second section assessed maternal knowledge of breastfeeding through 11 dichotomous items coded as “Yes"=1, “No"=0, and “Don’t know"=0. These items addressed key breastfeeding concepts, such as the benefits of breast milk, timing of initiation, duration of exclusive breastfeeding, the importance of colostrum, feeding during maternal or infant illness, and common misconceptions regarding supplemental fluids. Items phrased negatively were reverse-coded during analysis to maintain internal consistency. The third section measured maternal attitudes toward breastfeeding using 8 items rated on a 5-point Likert scale (1 = strongly disagree to 5 = strongly agree), evaluating emotional, practical, and social dimensions of maternal perception. Negatively worded statements were reverse-coded to ensure scoring validity. Scoring thresholds were defined to categorize levels of knowledge and attitudes. For the knowledge scale, total scores were interpreted as low (< 60% correct responses), moderate (60–79%), and high (≥ 80%). For the attitude scale, scores were categorized as negative (< 60%), neutral (60–79%), and positive (≥ 80%), consistent with previous maternal health research benchmarks. To ensure linguistic and cultural validity, the original English version of the instrument was translated into Kurdish using a rigorous forward–backward translation process. A pilot study involving 25 mothers was conducted to evaluate the clarity, cultural appropriateness, and comprehensibility of the items; data from this pilot sample were excluded from the final analysis. Reliability testing of the main study instrument demonstrated satisfactory internal consistency, with Cronbach’s alpha coefficients of 0.79 for the knowledge section and 0.84 for the attitude section, which are considered acceptable for public health research.

### Data collection

Data were collected exclusively through in-person interviews by a well-trained team of trained female research assistants using a questionnaire administered in the Kurdish language. Interviews were conducted face-to-face in private, culturally appropriate spaces within hospitals, healthcare centers, and affiliated clinics to ensure participant comfort, confidentiality, and open communication. Supervisory oversight and routine spot-check were conducted throughout the data collection to ensure protocol adherence, verify data accuracy, and address any procedural issues promptly. Social desirability bias was reduced through anonymity assurances and neutral language in the questionnaire.

### Statistical analysis

All collected data were analysed using Statistical Package for the Social Sciences (SPSS) version 26 (IBM, Corp., Armonk, NY, USA). Descriptive statistics were calculated and presented as frequencies, percentages, means, and standard deviations. Bivariate associations between sociodemographic variables and levels of knowledge and attitudes were examined using the Chi-square test (χ²). Ordinal logistic regression was used in univariate models to determine the predictors of high knowledge and positive attitudes. Multivariate ordinal logistic regression models were then constructed to adjust for potential confounding variables. Results were reported as adjusted odds ratios (Aor) with 95% confidence intervals (CI), and statistical significance was determined at *p* < 0.05. All assumptions for regression analysis, including multicollinearity and goodness-of-fit, were checked prior to final model interpretation.

## Results

### Sociodemographics of participants

Participants were predominantly aged 25–32 years (36.4%, *n* = 150), and nearly half (45.9%, *n* = 189) had attained education at the institute or college level. A majority resided outside the city (68.9%, *n* = 284), and over two-thirds (65.3%, *n* = 289) identified as housewives. Income distribution revealed that (35.2%, *n* = 145) earned between 400,000 and 900,000 Iraqi dinars (IQD) monthly. Multigravida mothers comprised two-third (67%, *n* = 276) and 40.5% (*n* = 167) had 2–3 living children. While 40.2% (*n* = 166) received breastfeeding education from relatives, only 23.8% (*n* = 98) were counselled by healthcare professionals (Table 1).

**Table 1:** Sociodemographics of enrolled participants

Variable	Frequency	Percentage
Age (Years)		
≤ 24	79	19.2
25–32	150	36.4
33–40	124	30.1
≥ 41	59	14.3
Level of education		
Illiterate	40	9.7
Primary school	153	37.1
Institute or college	189	45.9
Postgraduate	30	7.3
Residential area		
Outside the city	284	68.9
Inside the city	128	31.1
Occupation status		
Government employee	106	25.7
Private employee	20	4.9
Housewife	289	65.3
Others	17	4.1
Monthly income (Iraqi Dinar)		
100,000–600,000	74	18
400,000–600,000	145	35.2
700,000–900,000	118	28.6
> 1000,000	75	18.2
Pregnancy status		
Primigravida	136	33
Multigravida	276	67
Number of living children		
≤ 1	148	35.9
2–3	167	40.5
4–5	75	18.2
≥ 6	22	5.3
Received breastfeeding education during antenatal care		
No		
Yes-from healthcare professionals	98	23.8
Yes-from relatives	166	40.2
Yes-from online	41	10

### Knowledge toward breastfeeding

Nearly all participants recognized breastfeeding’s immunological advantages (98.3%, *n* = 405) and its role in enhancing maternal-child bonding (97.8%, *n* = 403). A high proportion (96.6%, *n* = 398) acknowledged its economic utility than formula feeding. However, critical gaps persisted, as 31.1% (*n* = 128) erroneously endorsed discontinuing breastfeeding during maternal or infant illness and 37.6% (*n* = 155) supported unnecessary fluid supplementation for exclusively breastfed infants. Only 68.9% (*n* = 284) correctly affirmed that breastfeeding not be interrupted during illness, while 76.7% (*n* = 316) adhered to recommendations against introducing complementary foods or fluids before 6 months (Table 2).

**Table 2:** Descriptive responses of mothers’ knowledge

Variable	Correct	Incorrect
	Frequency (%)	
Is breast milk good for infants’ immune systems?	405 (98.3)	7 (1.7)
Does breastfeeding increase maternal and child bonding?	403 (97.8)	9 (2.2)
Is breastfeeding convenient and cheaper economically?	398 (96.6)	14 (3.4)
Does breastfeeding help mothers to recover after delivery?	336 (81.6)	76 (18.4)
Is breastfeeding a well-balanced nourishing food?	377 (91.5)	35 (8.5)
Does breastfeeding help with infant teeth development?	353 (85.7)	59 (14.3)
Does breast milk fill up the stomach easily?	332 (80.6)	80 (19.4)
Does breastfeeding need to be stopped when the baby and/or mother is sick?	284 (68.9)	128 (31.1)
Should clear fluid be given to babies who are exclusively breastfeeding?	257 (62.4)	155 (37.6)
Is colostrum do good?	387 (93.9)	25 (6.1)
Foods or fluids should not be given to the baby under 6 months’ child age.	316 (76.7)	96 (23.3)

### Attitudes toward breastfeeding

**Fig. 1:**
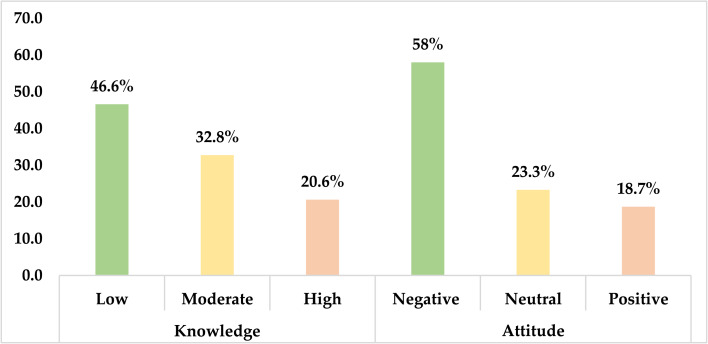
Levels of knowledge and attitude among respondents

Majority (72.3%, *n* = 298) of mothers agreed that breastfeeding was more convenient than formula feeding, and 60.4% (*n* = 249) affirmed its feasibility in public settings. However, nearly a quarter (24.8%, *n* = 102) perceived breastfeeding as detrimental to marital relationships and 33.5% (*n* = 138) reported to cease breastfeeding if discouraged by their spouse. Community support for breastfeeding was limited, with only 26% (*n* = 107) agreeing or strongly agreeing that their social environment encouraged the practice (Table 3). Regarding the distribution of knowledge and attitudes levels, only 20.6% of participants demonstrated high breastfeeding knowledge and 18.7% exhibited unequivocally positive attitudes (Fig. 1).

**Table 3:** Descriptive responses of mothers’ attitude

Variable	Strongly agree	Agree	Neutral	Disagree	Strongly disagree
	Frequency (%)				
Breastfeeding is easier than feeding infants formula.	298 (72.3)	65 (15.8)	32 (7.8)	12 (2.9)	5 (1.2)
Does breastfeeding have a negative effect on marital relationships?	40 (9.7)	51 (12.4)	88 (21.4)	131 (31.8)	102 (24.8)
Do breastfeeding mothers have difficulty taking care of their families?	132 (32)	74 (18)	67 (16.3)	89 (21.6)	50 (12.1)
Breastfeed babies with mother can give anywhere.	249 (60.4)	102 (24.8)	31 (7.5)	19 (4.6)	11 (2.7)
Can you start breastfeeding straight after delivery?	250 (60.7)	107 (26)	30 (7.3)	18 (4.4)	7 (1.7)
Mothers intending to breastfeed should expect sore nipples as a normal part of breastfeeding.	198 (48.1)	112 (27.2)	67 (16.3)	21 (5.1)	14 (3.4)
Does your community encourage breastfeeding?	107 (26)	36 (8.7)	54 (13.1)	96 (23.3)	119 (28.9)
Should you stop breastfeeding if your husband discourages?	42 (10.2)	36 (8.7)	61 (14.8)	135 (32.8)	138 (33.5)

### Associations between sociodemographic variables and levels of knowledge and attitude

Significant associations between sociodemographics and breastfeeding knowledge/attitudes were identified. Higher educational attainment correlated strongly with improved knowledge (χ²=21.044, *p* = 0.002) and positive attitudes (χ²=15.08, *p* = 0.020), with postgraduates demonstrating the highest knowledge (20% high knowledge vs. 12.5% among illiterates). Multigravida mothers exhibited superior knowledge (21.4% high vs. 19.1% in primigravida; χ²=6.47, *p* = 0.039) and more favorable attitudes (22.1% vs. 11.8%; χ²=10.01, *p* = 0.007). Conversely, higher-income mothers (> 1,000,000 IQD) reported disproportionately negative attitudes (70.7% negative vs. 50.3% in lower-income groups; χ²=12.64, *p* = 0.049). Guidance from healthcare professionals predicted higher knowledge retention (22.4% high vs. 15.9% in mothers without formal education; χ²=13.04, *p* = 0.042) (Table 4).

**Table 4:** Association between sociodemographic variables and levels of breastfeeding knowledge and attitude

Items	Knowledge Frequency (%)					Attitude Frequency (%)				
	Low	Moderate	High	χ2	*p*-value	Negative	Neutral	Positive	χ2	*p*-value
Age (Years)										
≤24	42(53.2)	24(30.4)	13(16.5)	7.953	.242	57(72.2)	14(17.7)	8(10.1)	9.749	.134
25-32	78(52)	41(27.3)	31(20.7)			78(52)	41(27.3)	31(20.7)		
33-40	49.5(39.5)	48(38.7)	27(21.8)			71(57.3)	28(22.6)	25(20.2)		
≥41	23(39)	22(37.3)	14(23.7)			33(55.9)	13 (22)	13(22)		
Level of education										
Illiterate	24(60)	11(27.5)	5(12.5)	6.123	.410	23(57.5)	6(15)	11(27.5)	21.044	.002*
Primary School	75(49)	47 (30.7)	31(20.3)			71(46.4)	45(29.4)	37(24.2)		
Institute or college	82(43.4)	64 (33.9)	43(22.8)			121 (64)	42(22.2)	26(13.8)		
Postgraduate	11(36.7)	13 (43.3)	6(20)			24(80)	3(10)	3(10)		
Residential area										
Outside the city	134 (47.2)	94 (33.1)	56(19.7)	0.466	.792	168(59.2)	62(21.8)	54(19)	1.106	.575
Inside the city	58(45.3)	41(32)	29(22.7)			71(55.5)	34(26.6)	23(18)		
Occupational status										
Governmental employee	36 (34)	44 (41.5)	26(24.5)	12.436	.053*	69(65.1)	25(23.6)	12(11.3)	15.079	.020*
Private employee	12 (60)	5 (25)	3 (15)			16(80)	2(10)	2(10)		
Housewife	137 (50.9)	78(29)	54(20.1)			141(52.4)	66(24.5)	62(23)		
Other	7 (41.2)	8 (47.1)	2 (11.8)			13(76.5)	3(17.6)	1(5.9)		
Monthly income (Iraqi Dinars)										
100,000-300,000	33 (44.6)	26 (35.1)	15(20.3)	1.759	.940	44(59.5)	17(23)	13(17.6)	12.643	.049*
400,000-600,000	67(46.2)	46(31.7)	32(22.1)			73(50.3)	41(28.3)	31(21.4)		
700,000-900,000	56 (47.5)	36 (30.5)	26 (22)			69(58.5)	22(18.6)	27(22.9)		
>1000,000	36 (48)	27 (36)	12 (16)			53(70.7)	16(21.3)	6(8)		
Pregnancy status										
Prim gravida	75(55.1)	35(25.7)	26(19.1)	6.470	.039*	93(68.4)	27(19.9)	16(11.8)	10.010	.007*
Multi gravida	117(42.4)	100(36.2)	59(21.4)			146(52.9)	69 (25)	61(22.1)		
Number of living children										
<=1	80(54.1)	37 (25)	31(20.9)	8.935	.177	103(69.6)	28(18.9)	17(11.5)	20.338	.002*
2-3	67(40.1)	64 (38.3)	36(21.6)			86(51.5)	44(26.3)	37(22.2)		
4-5	33(44)	28 (37.3)	14(18.7)			35(46.7)	23(30.7)	17(22.7)		
≥6	12(54.5)	6 (27.3)	4 (18.2)			15(68.2)	1(4.5%)	6(27.3)		
Received breastfeeding education										
No	63(58.9)	27 (25.2)	17(15.9)	13.042	.042*	61(57)	24(22.4)	22(20.6)	7.930	.243
Yes-from healthcare professionals	42(42.9)	34(34.7)	22(22.4)			51(52)	27(27.6)	20(20.4)		
Yes-from relatives	64 (38.6)	62 (37.3)	40(24.1)			106(63.9)	37(22.3)	23(13.9)		
Yes-from online	23(56.1)	12 (29.3)	6(14.6)			21(51.2)	8(19.5)	12(29.3)		

^*^Significant difference

### Factors predicting high breastfeeding knowledge and positive attitudes

Univariate regression analysis showed that women aged ≤ 24 years (COR = 0.369; 95% CI: 0.139–0.981; *p* = 0.046) and 25–32 years (COR = 0.438; 95% CI: 0.196–0.979; *p* = 0.044) were significantly less likely to have high breastfeeding knowledge compared to those aged ≥ 41 years. A positive attitude was significantly more common among women with primary school education (COR = 4.303; 95% CI: 1.296–14.280; *p* = 0.017) and those with a monthly income of 400,000–600,000 IQD (COR = 1.972; 95% CI: 1.049–3.707; *p* = 0.035). In contrast, not receiving breastfeeding education (COR = 0.379; 95% CI: 0.174–0.827; *p* = 0.015) and receiving it from relatives (COR = 0.345; 95% CI: 0.167–0.715; *p* = 0.004) had associated with significantly lower odds of a positive attitude (Table 5).

**Table 5:** Univariate ordinal logistic regression analysis to evaluate the predictive factors associated high knowledge, and positive attitude toward breastfeeding (*n*=412)

Items	High Level of Knowledge				Positive Attitude			
	COR	95% CI		*p*-value	COR	95% CI		*p*-value
Age in years (ref = 41+)								
≤ 24	0.369	0.139	0.981	0.046^*^	0.459	0.166	1.270	0.134
25–32	0.438	0.196	0.979	0.044^*^	1.203	0.536	2.697	0.654
33–40	0.611	0.306	1.219	0.162	1.025	0.501	2.095	0.946
Level of education (ref=postgraduate)								
Illiterate	0.352	0.100	1.242	0.105	3.287	0.784	13.769	0.104
Primary school	0.938	0.358	2.459	0.896	4.303	1.296	14.280	0.017^*^
Institute or college	1.070	0.483	2.369	0.868	2.108	0.741	5.994	0.162
Occupational status (ref=others)								
Governmental employee	1.012	0.351	2.914	0.983	1.008	0.271	3.749	0.991
Private employee	0.539	0.148	1.956	0.347	0.522	0.101	2.698	0.438
Housewife	0.778	0.297	2.037	0.610	1.242	0.362	4.254	0.731
Residential area (ref=inside of the city)								
Outside of the city	0.790	0.513	1.218	0.286	1.116	0.709	1.755	0.636
Monthly income in Iraqi dinars (ref = > 1000,000)								
100,000-300,0000	1.709	0.858	3.405	0.127	1.589	0.750	3.363	0.226
400,000-600,000	1.604	0.897	2.871	0.111	1.972	1.049	3.707	0.035^*^
700,000-900,000	1.290	0.718	2.319	0.395	1.844	0.963	3.530	0.065
Pregnancy status (ref=multigravida)								
Primigravida	0.614	0.276	1.365	0.232	1.117	0.448	2.787	0.812
Number of livings children (ref = ≥ 6+)								
<=1	1.476	0.357	6.110	0.591	0.972	0.205	4.597	0.971
2–3	1.152	0.357	3.723	0.813	1.950	0.548	6.942	0.303
4–5	0.886	0.291	2.696	0.831	1.937	0.584	6.431	0.280
Receiving breastfeeding education (ref=online)								
No	0.898	0.422	1.910	0.780	0.379	0.174	0.827	0.015*
Yes-from healthcare professionals	1.517	0.724	3.175	0.269	0.483	0.223	1.044	0.064
Yes-from relatives	1.762	0.890	3.492	0.104	0.345	0.167	0.715	0.004*

*OR* Odds ratio, *CI* Confidence interval, *vs*. versus

* Significant difference

Multivariate regression analysis revealed that women aged ≤ 24 years (Aor = 0.430; 95% CI: 0.187–0.985; *p* = 0.046) and 25–32 years (Aor = 0.462; 95% CI: 0.234–0.910; *p* = 0.025) remained significantly less likely to have high breastfeeding knowledge compared to those aged ≥ 41 years. Illiterate women were also significantly less likely to have high breastfeeding knowledge (Aor = 0.280; 95% CI: 0.099–0.789; *p* = 0.016) and demonstrated higher odds of positive breastfeeding attitudes than women with postgraduate education. Similarly, only those aged ≤ 24 years (Aor = 0.311; 95% CI: 0.140–0.690; *p* = 0.004) were significantly less likely to report positive breastfeeding attitudes. Conversely, compared with postgraduate mothers, illiterate women were significantly more likely to report positive breastfeeding attitudes (Aor = 3.567; 95% CI: 1.051–12.105; *p* = 0.041) (Table 6). Furthermore, several regression estimates showed relatively wide confidence intervals, suggesting instability of some associations and reduced statistical precision. Therefore, the observed associations should be interpreted with caution.

**Table 6:** Factors predicting high breastfeeding knowledge and positive attitude based on multivariate ordinal logistic regression analysis

Items	High Level of Knowledge				Positive Attitude			
	AOR	95% CI		*p*-value	AOR	95% CI		*p*-value
Age in years (ref = 41+)								
≤ 24	0.430	0.187	0.985	0.046*	0.311	0.140	0.690	0.004*
25–32	0.462	0.234	0.910	0.025*	1.095	0.564	2.127	0.789
33–40	0.645	0.0340	1.223	0.179	1.142	0.584	2.231	0.698
Level of education (ref=postgraduate)								
Illiterate	0.280	0.099	0.789	0.016*	3.567	1.051	12.105	0.041*
Primary school	0.738	0.327	1.667	0.465	5.971	2.085	17.102	0.001*
Institute or college	0.914	0.430	1.943	0.816	2.328	0.851	6.369	0.100
Occupational status (ref=others)								
Governmental employee	-	-	-	-	-	-	-	-
Private employee	-	-	-	-	-	-	-	-
Housewife	-	-	-	-	-	-	-	-
Residential area (ref=inside of the capital city)								
Outside of the city	-	-	-	-	-	-	-	-
Monthly income in Iraqi dinar (ref = > 1000,000)								
100,000-300,0000	1.740	0.889	3.405	0.106	1.569	0.751	3.279	0.231
400,000-600,000	1.598	0.901	2.832	0.109	1.937	1.038	3.615	0.038*
700,000-900,000	1.275	0.718	2.266	0.407	1.853	0.977	3.513	0.059
Pregnancy status (ref=multigravida)								
Primigravida	0.756	0.456	1.253	0.277	-	-	-	-
Number of livings children (≥ 6)								
≤ 1	-	-	-	-	-	-	-	-
2–3	-	-	-	-	-	-	-	-
4–5	-	-	-	-	-	-	-	-
Received breastfeeding education (ref=online)								
No	0.886	0.420	1.870	0.751	0.373	0.173	0.803	0.012*
Yes-from healthcare professionals	1.559	0.755	3.222	0.230	0.492	0.231	1.047	0.066
Yes-from relatives	1.769	0.897	3.487	0.099	0.351	0.172	0.719	0.004*

*AOR* Adjusted odds ratio, *CI* Confidence interval, *vs*. versus

^*^Significant difference

^*^Variables with dashes were excluded from the final multivariate model due to non-significance in adjusted analysis

## Discussion

This study found that only 20.6% of mothers in the Kurdistan Region, Iraq demonstrated high levels of breastfeeding knowledge, while just 18.7% held a clearly positive attitude toward breastfeeding. Younger mothers (≤ 24 years), illiterate with primary school, and those who received breastfeeding information from non-professional sources were significantly less likely to have either high knowledge or favourable attitudes. The lower breastfeeding knowledge observed among some mothers may partially reflect persistent community misconceptions regarding breastfeeding benefits, milk adequacy, and infant feeding practices. Traditional myths may continue to affect maternal attitudes despite increasing access to healthcare information. Healthcare providers should actively address breastfeeding misconceptions through culturally sensitive education programs targeting both mothers and family members. Unexpectedly, illiterate mothers showed higher odds of positive breastfeeding attitudes than postgraduate women. This finding may reflect stronger adherence to traditional breastfeeding practices among less-educated women, whereas highly educated mothers may experience greater occupational pressures, formula-feeding exposure, or lifestyle-related barriers. The findings of the current study should be interpreted within the Kurdish sociocultural context, where family-centred decision-making and traditional maternal practices remain influential. In Kurdish communities, breastfeeding attitudes are not determined solely by maternal education or healthcare counselling but are also shaped by cultural expectations and family experiences. The findings should also be interpreted within the broader post-conflict healthcare context of Iraq, where healthcare resources and maternal support services may be limited. Long-term healthcare system strain may reduce opportunities for comprehensive breastfeeding counselling and maternal education. Despite these challenges, positive breastfeeding attitudes observed among some mothers may reflect the cultural normalization of breastfeeding within Kurdish communities. Notably, moderate-income mothers (400,000–600,000 IQD monthly) were more likely to hold positive attitudes. These findings reveal substantial educational and structural disparities in breastfeeding awareness and support, with implications for maternal and child health outcomes in the region. The observed gaps in breastfeeding knowledge may indicate deficiencies in antenatal and postnatal breastfeeding education within healthcare facilities. Inconsistent counselling practices and insufficient breastfeeding support programs may contribute to inadequate maternal awareness. Strengthening breastfeeding education within primary healthcare canters and maternity hospitals may improve maternal knowledge and breastfeeding outcomes. The findings align with global trends in which maternal education and age are key determinants of breastfeeding-related knowledge and behaviour. Similar studies in low- and middle-income countries have found that younger and less-educated mothers tend to have limited awareness of breastfeeding benefits and are less likely to adhere to exclusive breastfeeding practices [12, 13]. In Nepal, Dharel et al. observed that maternal low education levels were strongly associated with suboptimal breastfeeding practices [5]. These results also align with Sharma and Byrne (2016), which emphasized that early initiation of breastfeeding and sustained exclusivity are strongly associated with maternal age and educational attainment [16]. Likewise, Kavle et al. noted that cultural misconceptions and limited professional support remain pervasive barriers in similar settings [17].

Regionally, our findings corroborate previous work in Iraq and neighbouring Middle Eastern countries. For instance, Ameer et al. identified widespread breastfeeding among Iraqi mothers, but also documented significant knowledge deficits, particularly exclusive breastfeeding and the importance of colostrum [18]. A data from Erbil by Ahmed and Piro (2019), similarly found high breastfeeding intent but poor knowledge of evidence-based practices. In Duhok, exclusive breastfeeding rates were as low as 7.4%, despite maternal enthusiasm. Such inconsistencies between attitude and behaviour suggest systemic gaps in health education and support, especially in the semi-autonomous Kurdish region [14].

Interestingly, the role of income in shaping breastfeeding attitudes in this study deviated slightly from conventional expectations. While global data often link higher income to better health behaviours. This study revealed that middle-income mothers had more favourable breastfeeding attitudes than their higher-income counterparts. This mirrors findings from previous studies, where higher socioeconomic status was sometimes associated with early cessation of breastfeeding due to work commitments and social preferences for formula feeding [19–21]. These findings may be understood of sociocultural norms, healthcare access, and information sources. In many communities, informal knowledge networks, such as advice from relatives; often substitute formal antenatal education, which may perpetuate misconceptions, such as the belief that colostrum is harmful or infants need water in hot weather.

Additionally, this study found that illiteracy significantly reduced both knowledge and attitudes scores. This is consistent with the literature indicating that maternal education not only improves health literacy, but also enhances receptivity to evidence-based health messages [12, 22]. The weak influence of healthcare professionals in shaping attitudes is concerning. Only 23.8% of participants received breastfeeding education from clinical sources. Given that professional counselling is positively associated with improved outcomes. This underutilization of healthcare resources may reflect both systemic limitations and cultural barriers to accessing formal antenatal education [3, 6].

This study also suggest that parity plays a role, as multigravida women had significantly higher knowledge and more favourable attitudes compared to primigravida. This trend may reflect learning through experience, underscoring the importance of early antenatal education for first-time mothers. Nearly half of mothers incorrectly believed that fluids should be given to exclusively breastfed infants, and > 31% thought breastfeeding should be stopped during maternal or infant illness. These findings point to persistent myths that undermine optimal breastfeeding, the regression analysis supports this, indicating that non-healthcare sources of education; especially relatives; were associated with significantly lower odds of a positive breastfeeding attitude as previously documents in several studies [23, 24]. This study highlights an urgent need for integrating structured, culturally sensitive breastfeeding education into routine antenatal and postnatal care in the Kurdistan Region. Healthcare professionals must play a more active role, with breastfeeding counselling embedded into the maternal health continuum, particularly for younger and less-educated mothers. Education should include myth-busting modules on colostrum, feeding during illness, and the sufficiency of breast milk without additional fluids. In-service training for midwives and nurses, expansion of Baby-Friendly Hospital Initiatives [25]. Policy-makers should consider mandating lactation education within public health frameworks, aligned with the WHO Global Breastfeeding Collective’s 2025 targets. Furthermore, engaging fathers and elder women in educational initiatives may improve family support systems and reduce the impact of conflicting advice. Public health campaigns using culturally appropriate messaging, delivered in the Kurdish language via radio, TV, and social media, can also help shift public perceptions. This study’s strengths include a large and regionally representative sample, use of validated tools, and robust statistical modelling. However, limitations exist, such as its cross-sectional design which precludes causal inference, and the use of self-reported measures introduces the risk of social desirability and recall biases [14]. The non-probability purposive sampling method may limits statistical generalizability of our findings to the broader population of Iraq or all Kurdish mothers nationwide, though efforts were made to include diverse healthcare settings [25]. However, to mitigate this limitation and minimize selection bias, participants were recruited across a diverse mix of both public and private maternal healthcare facilities, ensuring a wide representation of socioeconomic backgrounds.

## Conclusions

This study highlights substantial gaps in breastfeeding knowledge and attitudes among mothers in the Kurdistan Region, with younger and less-educated women being most affected. Illiteracy and informal sources of breastfeeding education were associated with poorer outcomes, while moderate income levels showed a positive influence on attitudes. These findings underscore the urgent need for targeted, professional breastfeeding education integrated into maternal healthcare services. Cultural interventions and strengthened policy support are essential to promote optimal breastfeeding practices and improve maternal and child health in the region. Future studies should consider longitudinal designs to evaluate the impact of structured breastfeeding interventions over time. Qualitative research exploring the sociocultural dynamics behind breastfeeding choices, such as the influence of fathers, grandmothers, and social norms may provide deeper understanding. Additionally, implementation trials testing various modes of breastfeeding education delivery (e.g. digital tools and community health workers) would be valuable in shaping effective policy.

## Data Availability

The datasets used and/or analysed during the current study available from the corresponding author and first author on reasonable request.

## Abbreviations

Aor: Adjusted Odds Ratio
BFHI: Baby-Friendly Hospital Initiative
COR: Crude Odds Ratio
CI: Confidence Interval
EBF: Exclusive Breastfeeding
IQD: Iraqi Dinar
IBM: International Business Machines Corporation
IRB: Institutional Review Board
χ²: Chi-square test
SPSS: Statistical Package for the Social Sciences
STROBE: Strengthening the Reporting of Observational Studies in Epidemiology
SD: Standard Deviation
UNICEF: United Nations Children’s Fund
WHO: World Health Organization
